# Therapeutic Effect of Matrine on Collagen-Induced Arthritis Rats and Its Regulatory Effect on RANKL and OPG Expression

**DOI:** 10.1155/2021/4186102

**Published:** 2021-08-09

**Authors:** Xin Li, Lijie Zhang, Yongsong Xu, Dong Zhao, Lin Mu, Baoyu Zhang, Jing Ke

**Affiliations:** ^1^Center for Endocrine Metabolism and Immune Diseases, Beijing Luhe Hospital, Capital Medical University, Beijing 101149, China; ^2^Beijing Key Laboratory of Diabetes Research and Care, Beijing 101149, China

## Abstract

**Objective:**

To investigate the effect of matrine on rats with collagen-induced arthritis (CIA) and its regulatory effect on receptor activator of nuclear factor-*κ*B ligand (RANKL) and osteoprotegerin (OPG) expression.

**Methods:**

Wistar rats (*n* = 6) and CIA rats (*n* = 30) were randomly divided into six groups: healthy, CIA control, low/medium/high matrine (25, 50, or 100 mg/kg, once per day for six weeks), and methotrexate (MTX) (2 mg/kg, once per week for six weeks). The degree of joint damage was evaluated by X-ray and HE staining. Bone marrow suppression was assessed by routine blood analysis. In addition, the levels of serum RANKL and OPG in the rats were measured by ELISA.

**Results:**

The level of joint swelling and degree of joint damage assessed by ankle swelling measurements, AI score, X-ray, and HE staining were alleviated in the CIA rats treated with MTX or different doses of matrine. Furthermore, no obvious inhibitory effect was observed on the bone marrow of the CIA rats, regardless of the dose of matrine or treatment with 2 mg/kg MTX (*P* > 0.05). The levels of OPG in serum and the ratio of OPG/RANKL were higher, and RANKL expression was lower in the low/medium/high matrine group compared with that of the CIA control group. The serum levels of OPG and OPG/RANKL ratio increased with the matrine dose, while the opposite was observed for RANKL expression.

**Conclusion:**

Matrine treatment was associated with a lower degree of bone destruction, increased OPG expression and OPG/RANKL ratio, and decreased RANKL expression in CIA rats. Thus, matrine may represent a novel drug candidate for the treatment of RA.

## 1. Introduction

Rheumatoid arthritis (RA) is an autoimmune disease characterized by symmetrical multijoint bone destruction, accompanied by a high disability and mortality rate. While decreased bone mass and bone destruction around the joints represent the main causes of disability in RA patients, no effective treatment is currently available to reverse or repair RA-induced cartilage and bone destruction.

Osteoclasts are the only multinuclear terminal differentiated cells in the human body that have the ability to destroy bone [1]. As such, osteoclasts play an indispensable role in maintaining the process of bone resorption and bone formation [2]. Recent research into the mechanism of osteoclast activation has resulted in important breakthroughs in the search for novel targets for RA treatment.

Increased secretion of receptor activator of nuclear factor-*κ*B ligand (RANKL) is required for osteoclast differentiation [1]. RANKL has two related receptors. The first is NF-*κ*B receptor activator of nuclear factor-*κ*B (RANK), which is mainly located on the cell membrane surface of osteoclast precursor cells. RANKL can bind to RANK to activate intracellular-related signaling pathways, which can promote osteoclastogenesis. The other receptor is osteoprotegerin (OPG), which is secreted by osteoblasts and exists in the extracellular matrix. Moreover, OPG can compete with RANK to inhibit osteoclast differentiation [3, 4]. The RANKL/RANK/OPG system is important for regulating bone formation and bone destruction. The RANKL/OPG ratio can determine the direction of bone change; a higher RANKL/OPG ratio indicates an increase in bone loss, while a lower ratio is associated with decreased bone loss [5].

Many natural Chinese herbal medicines have been found to have an effect on the prevention and treatment of bone diseases. Moreover, studies have confirmed that some Chinese medicines can reduce RA-associated inflammation and inhibit pannus formation by modulating the signal transduction pathways involved in osteoclast production [6, 7]. There is a need to identify a new, effective, and less toxic drug for the treatment of RA.

Matrine is a major component of the traditional Chinese herb, *Sophora flavescens*, which has anti-inflammatory, antitumor, immunosuppressive, and other effects [8, 9]. In rats with type II collagen-induced arthritis (CIA), matrine has been shown to decrease the degree of joint swelling, arthritis index (AI), and the joint inflammatory response by reducing the level of proinflammatory factor expression, including tumor necrosis factor-*α* (TNF-*α*), interleukin- (IL-) 1*β*, IL-6, IL-8, IL-17A, matrix metalloproteinase- (MMP-) 2, MMP-3, and MMP-9 [10]. Matrine can also inhibit the expression of osteoclastogenesis-related biomarkers, such as MMP-9, nuclear factor of activated T cells 1 (NFATc1), TRAP, C-Src, and cathepsin K by inhibiting the activation of NF-*κ*B, AKT, and MAPK signaling pathways [11]. Similarly, we previously reported that matrine could inhibit osteoclast differentiation [12]. However, the detailed mechanism remains unknown. In this study, we show that matrine affects OPG and RANKL expression and plays a role in regulating osteoclast differentiation, providing treatment for RA.

## 2. Materials and Methods

### 2.1. Animals

Healthy six-week-old female Wistar rats (160 g–180 g) were purchased from the Beijing Vital River Laboratory Animal Technology Co. Ltd. (Beijing, China) and housed at the Beijing Key Laboratory of Diabetes Research and Care in a room temperature at 20°C and 50%-55% humidity. The rats were freely permitted to move, eat, and drink freely. The experimental protocols were approved by the Capital Medical University Institutional Animal Care and Use Committee (IACUC) (no. AEEI-2021-031) and compliant with the National Institutes of Health (NIH) laboratory animal protection and use guidelines.

### 2.2. Matrine

Matrine (no.1808232) was purchased from Shanxi Zhendong Pharmaceutical (Shanxi, China) with purity greater than 98%. The chemical formula is C_15_H_24_N_2_O; the molecular weight is 248.37. Matrine (solubility: 15 g/L) was dissolved in distilled water. The lethal dose 50 (LD50) of matrine that was injected intraperitoneally into rats was 125 mg/kg.

### 2.3. Preparation and Treatment of CIA Rats

Thirty-six Wistar rats were randomly divided into six groups: healthy, CIA control, methotrexate (2 mg/kg MTX group), low dosage matrine (25 mg/kg matrine group), medium dosage matrine (50 mg/kg matrine group), and high dosage matrine (100 mg/kg matrine group). Rats in the healthy group received 0.9% normal saline as a vehicle injection. The other 30 rats were used to establish a CIA model by immunizing healthy Wistar rats with 0.1 mL mixed emulsion twice (at three-week intervals). The first mixed emulsion consisted of 4 mL type II collagen (Chondrex, USA) and 4 mL Freund's complete adjuvant (CFA, Sigma, USA). The second mixed emulsion was composed of an equal volume of type II collagen and Freund's incomplete adjuvant (IFA, Chondrex, USA). After 22 days, the rats were intraperitoneally injected with an equal volume (2 mL) of 0.9% normal saline (healthy group and CIA control group), 25 mg/kg matrine, 50 mg/kg matrine, or 100 mg/kg matrine once daily, except for 2 mg/kg MTX (Pfizer (Perth) Pty Limited, Australia), for which treatment was administered once per week. Matrine and MTX were diluted with 0.9% normal saline. All groups received treatment for up to six weeks postimmunization.

### 2.4. Clinical Evaluation

Each rat was weighed, and the clinical severity of arthritis was evaluated weekly using a Vernier caliper and AI scoring. These procedures were performed by two independent observers. The AI score was determined as follows: 0: no swelling or erythema, 1: slight swelling or erythema of one toe, 2: moderate redness and swelling spread to lower limbs or swelling of more than two toes, 3: severe redness and swelling of lower limbs, and 4: joint rigidity or malformation. Each animal was rated on a scale of 0-16.

### 2.5. X-Ray Evaluation

The rats were anesthetized after treatment. The rats were subjected to an X-ray to observe the degree of bone destruction to the right toe joints and scored according to the Larsen scoring standard. The Larsen score was determined as follows: 0 points: normal; 1 point: soft tissue swelling around the joints, osteoporosis, and/or slight joint space stenosis; 2 points: bone erosion, with or without joint space stenosis; 3 points: mild joint destruction with less than 50% joint space stenosis; 4 points: severe joint destruction, more than 50% joint space stenosis, but no disappearance of the joint space; and 5 points: multiple joints were damaged and disappearance of the joint space. The Larsen score was jointly assessed by a radiologist and a rheumatologist.

### 2.6. Hematoxylin-Eosin Staining (HE Staining) Evaluation

After the rats were sacrificed, the right hind limbs were placed in a 10% neutral formalin solution for 10 days, and the fluid was changed every three days. They were decalcified with a 10% EDTA solution for two months. The samples were then dehydrated, paraffin-embedded, and sliced. The degree of bone destruction in the ankle joints was observed by HE staining.

### 2.7. Bone Marrow Suppression Was Assessed by Routine Blood Analysis

After six weeks of treatment, we evaluated matrine or MTX for bone marrow inhibition in CIA rats by performing a routine blood analysis. The test kits were purchased from Mindray Biomedical Electronics Co., Ltd. and the serum of rats was analyzed by a routine blood analyzer for animals (Mindray BC2800Vet, Shenzhen, China).

### 2.8. RANKL and OPG in the Serum Were Measured by an ELISA

The serum samples of the rats were stored at -80°C. When the samples underwent further analysis, they were thawed on ice and subsequently centrifuged for 20 min (1000 × g). The level of serum RANKL and OPG expression was measured using commercial ELISA kits (mlbio, Shanghai, China) in accordance with the manufacturer's instructions.

### 2.9. Statistical Analysis

The data were analyzed using SPSS22.0 software. The differences between the groups were analyzed by a one-way ANOVA. In addition, we compared the difference between any two groups using the Tukey method. The normal distribution variables were presented as the mean ± SEM. *P* < 0.05 was considered to be statistically significant.

## 3. Results

### 3.1. Clinical Effect of Matrine on CIA Rats

**Figure 1:**
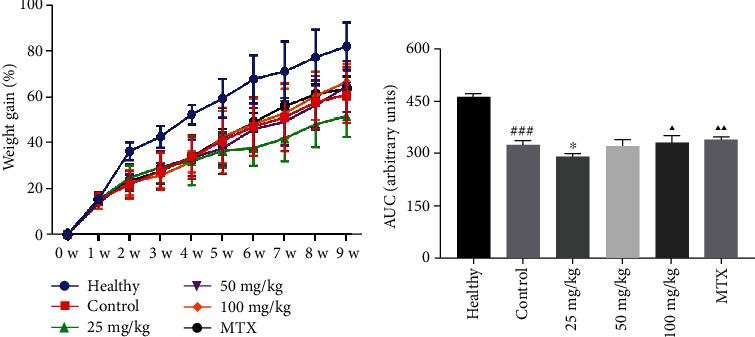
Changes in weight gain of the rats in each group. Healthy: healthy group; control: CIA control group; 25 mg/kg: low dosage matrine group; 50 mg/kg: medium dosage matrine group; 100 mg/kg: high dosage matrine group; MTX: 2 mg/kg methotrexate group. Compared to the healthy group, ^###^*P* < 0.001; compared with the control group, ^∗^*P* < 0.05; compared to the 25 mg/kg group, ^▲^*P* < 0.05; ^▲▲^*P* < 0.01.

**Figure 2:**
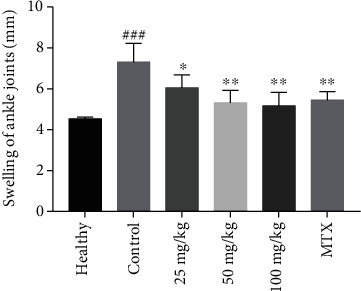
Therapeutic effects of matrine and MTX on swelling of ankle joints in CIA rats. Ankle joint swelling was evaluated after treatment. Healthy: healthy group; control: CIA control group; 25 mg/kg: low dosage matrine group; 50 mg/kg: medium dosage matrine group; 100 mg/kg: high dosage matrine group; MTX: 2 mg/kg methotrexate group. Compared to the healthy group, ^###^*P* < 0.001; compared to the control group, ^∗^*P* < 0.05; ^∗∗^*P* < 0.01.

Wistar rats were induced into CIA rats by immunization with type II collagen. We observed that the redness and swelling of the joints were more severe (*P* < 0.001), the AI score was higher (*P* < 0.001), and the weight gain was lower in the CIA rats compared to the healthy rats (*P* < 0.05). After treating the rats for six weeks, we observed weight gain as shown in Table 1 and Figure 1. Compared with the CIA control group (4.53 ± 0.082), the swelling of ankle joints in the 25 mg/kg matrine (6.05 ± 0.641) (*P* < 0.05), 50 mg/kg matrine (5.32 ± 0.599) (*P* < 0.01), 100 mg/kg matrine (5.16 ± 0.668) (*P* < 0.01), and 2 mg/kg MTX (5.45 ± 0.411) (*P* < 0.01) groups was lower (Figure 2). Similar results were obtained from the AI score (Table 2).

### 3.2. Matrine Can Decrease the Degree of Joint Damage Evaluated by X-Ray and HE Staining

**Figure 3:**
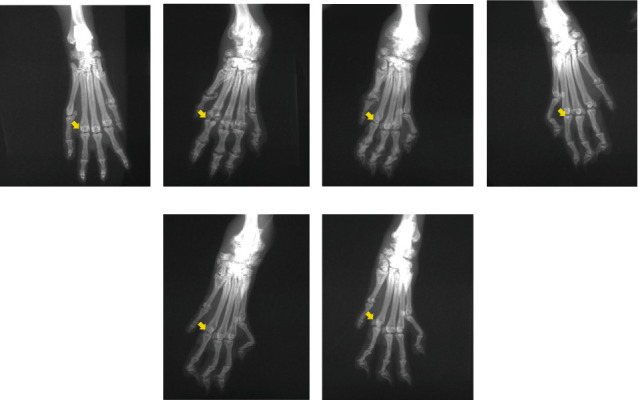
X-ray was used to evaluate the therapeutic effects of matrine and MTX on CIA rats: (a) healthy group; (b) CIA control group; (c) low dosage matrine group (25 mg/kg matrine group); (d) medium dosage matrine group (50 mg/kg matrine group); (e) high dosage matrine group (100 mg/kg matrine group); (f) methotrexate group (2 mg/kg MTX group). Obvious joint damage and part of the joint space disappearance could be observed in (b). In (c–f), the degree of joint damage was lower than that in (b).

We evaluated the degree of joint damage by X-ray (Figure 3) and Larsen scoring. The X-ray clearly revealed bone destruction in the toe joints of CIA rats. Following treatment, the Larsen scores of the 50 mg/kg matrine (2.50 ± 1.049), 100 mg/kg matrine (2.00 ± 1.414), and 2 mg/kg MTX (2.33 ± 1.033) groups were lower than that of the CIA control group (4.33 ± 0.817) (*P* < 0.05). However, there was no statistically significant difference in the Larsen score between the different treatment groups.

**Figure 4:**
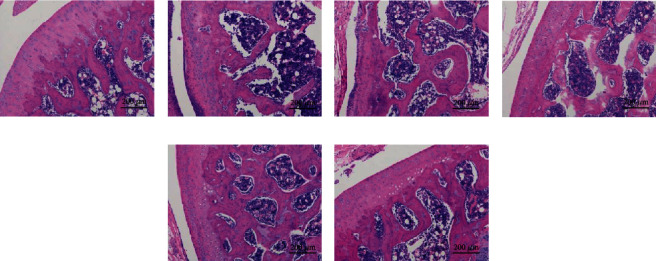
HE staining was used to evaluate the therapeutic effects of matrine and MTX on CIA rats. HE staining (magnification: 100x): (a) healthy group; (b) CIA control group; (c) low dosage matrine group (25 mg/kg matrine group); (d) medium dosage matrine group (50 mg/kg matrine group); (e) high dosage matrine group (100 mg/kg matrine group); (f) methotrexate group (2 mg/kg MTX group).

HE staining revealed complete articular cartilage and normal synovial tissue in the healthy group, without inflammatory cell infiltration. In the CIA control group, a large amount of inflammatory cell infiltration and synovial cell proliferation was observed around the ankle joints. Following treatment, the arrangement of synovial cells was relatively regular, and the proliferation of synovial cells and infiltration of inflammatory cells were significantly reduced in the 50 mg/kg matrine, 100 mg/kg matrine, and 2 mg/kg MTX groups (Figure 4).

### 3.3. Matrine Exhibited No Obvious Inhibitory Effect on the Bone Marrow of the CIA Rats

**Figure 5:**
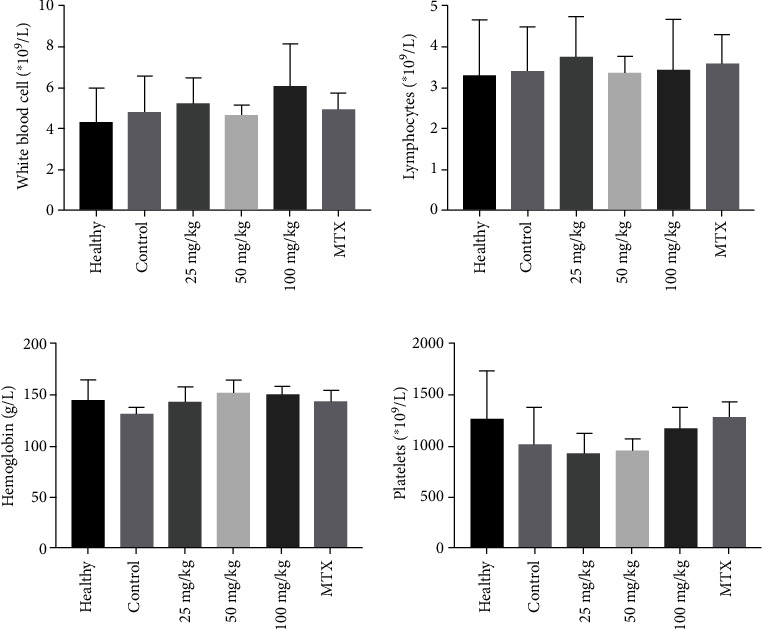
Effects of matrine and MTX on routine blood analysis of CIA rats. Serum concentrations of white blood cells, lymphocytes, hemoglobin, and platelets were tested after treatment: (a) white blood cells; (b) lymphocyte; (c) hemoglobin; (d) platelets. Healthy: healthy group; control: CIA control group; 25 mg/kg: low dosage matrine group; 50 mg/kg: medium dosage matrine group; 100 mg/kg: high dosage matrine group; MTX: 2 mg/kg methotrexate group.

Following treatment, we confirmed the inhibitory effect on the bone marrow in the CIA rats following different doses of matrine and 2 mg/kg MTX using a routine blood analysis. Interestingly, regardless of whether it was different doses of matrine or 2 mg/kg MTX, no obvious bone marrow inhibitory effect was observed in the CIA rats (*P* > 0.05) (Figure 5).

### 3.4. The Level of Serum RANKL and OPG Expression Assessment by ELISA

**Figure 6:**
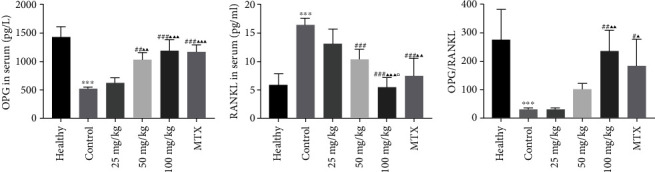
The level of OPG and RANKL expression in the serum assessed by an ELISA. (a) The level of OPG expression in the serum; (b) the level of RANKL expression in the serum; (c) the expression of OPG/RANKL. Healthy: healthy group; control: CIA control group; 25 mg/kg: low dosage matrine group; 50 mg/kg: medium dosage matrine group; 100 mg/kg: high dosage matrine group; MTX: 2 mg/kg methotrexate group. Compared to the healthy group, ^∗∗∗^*P* < 0.001; compared to the control group, ^#^*P* < 0.05, ^##^*P* < 0.01, and ^###^*P* < 0.001; compared to the 25 mg/kg group, ^▲▲^*P* < 0.01, ^▲▲▲^*P* < 0.001; compared to the 50 mg/kg group, ^□^*P* < 0.05.

After the rats were immunized with type II collagen, the level of OPG (523.25 ± 23.081) and OPG/RANKL ratio (32.20 ± 3.420) expression was decreased, whereas RANKL expression (16.32 ± 1.122) was significantly increased in the CIA control group compared with that in the healthy group (OPG: 1422.00 ± 174.091, OPG/RANKL: 275.03 ± 104.824, and RANKL: 5.80 ± 0.820) (*P* < 0.001). The lowest levels of OPG and OPG/RANKL ratio, as well as the highest levels of RANKL expression, were observed in the CIA control group compared to the other five groups. With treatment of different doses of matrine and 2 mg/kg MTX, the levels of serum OPG and OPG/RANKL ratio were higher, whereas the expression of RANKL was lower than that in nontreated CIA rats. We found that the levels of OPG and OPG/RANKL ratio expression increased with increasing doses of matrine, whereas the opposite was observed for the level of RANKL expression. The 50 mg/kg matrine and 100 mg/kg matrine groups exhibited a similar effect on the level of OPG and RANKL expression compared to the 2 mg/kg MTX group (*P* > 0.05) (Figure 6).

## 4. Discussion

RA is an autoimmune disease characterized by synovitis as the main pathological manifestation and symmetrical multijoint injury as the primary feature, which is highly disabling. RA seriously affects the physical and mental health of patients and also causes a substantial economic burden [13]. Besides genetic factors, *P. gingivalis* represents one of the main environmental factors that can lead to autoimmune arthritis, which can induce citrullinated peptides. Citrullinated peptides are a source of anticitrullinated antibodies (ACPAs), which can predict the occurrence and the future development of RA [14]. An increasing number of studies have found a correlation between *P. gingivalis* infection and ACPA-positive RA [15]. In addition, some studies have found that drug targeting *P. gingivalis* infection can effectively treat RA [16]. Recently, although an increasing number of drugs have been observed to be used for the treatment of RA, studies have shown that only some people could achieve sustained clinical remission [17, 18]. This phenomenon may be related to factors, such as drug side effects or the associated economic burden. Therefore, researchers have sought to identify novel drugs with few side effects and lower prices for the treatment of RA.

Matrine is an important ingredient of the legume plant, *Sophora flavescens*, which has a wide range of pharmacological effects and has been widely used in clinical practice for the treatment of lung, breast, esophageal, gastric, and liver cancers [19, 20]. Some studies have shown that matrine can effectively inhibit the inflammatory response [21, 22]. Yang and Wu [23] reported that matrine may effectively reduce the release of the terminal inflammatory factors, IL-1*β* and TNF-*α*, by inhibiting the overexpression of TLR4 and c-Jun in the protein kinase signaling pathway of LPS-induced macrophage mitogen activation, thereby effectively inhibiting the inflammatory response. In addition, matrine has also been found to exhibit immunosuppressive effects [9]. Research into gastric cancer has shown that sophoridine acts on macrophages and CD8^+^ T cells to reshape the immune microenvironment in gastric cancer [24]. Moreover, matrine has been demonstrated to be effective at inducing T cell anergy in human Jurkat cells [25]. In light of the immunosuppressive and anti-inflammatory effects associated with matrine, researchers have recently turned their attention to whether matrine can be used to treat autoimmune diseases.

In the treatment of RA, researchers have found that matrine can inhibit the inflammatory response in CIA rats [10]. Treatment with matrine in vitro was shown to reduce the level of Th1 cytokines (e.g., IFN-*γ*, TNF-*α*, and IL-1*β*) and increase the level of Th2 cytokines (e.g., IL-4 and IL-10) by reducing the NF-*κ*B signaling pathway in T cells. Therefore, matrine regulated the Th1 and Th2 cytokine imbalance in RA rats [26]. MASM, a matrine derivate, can inhibit the expression of proinflammatory cytokines (e.g., TNF-*α*, IL-6, and IL-8) and MMP-1, MMP-3, and MMP-13 by blocking MAPK phosphorylation and NF-*κ*B activation in vitro. In addition, MASM was found to induce apoptosis in RA fibroblast-like synoviocytes (FLS) [27]. In the same year, Yang et al. [28] published similar results, reporting that matrine could induce the apoptosis of FLS in CIA rats by inhibiting activation of the JAK/STAT signaling pathway. In addition, the study by Chen et al. [29] found that the inhibitory effect of matrine on the growth and cell cycle of peripheral lymphocytes from RA patients was comparable to that of MTX. Matrine could increase the ratio of cells in the G0/G1 phase and decrease the ratio of cells in the S phase and C2/M state in a dose-dependent manner. Therefore, we also focused on the therapeutic effect of matrine on CIA rats. In contrast to the findings of previous studies, we first observed the therapeutic effect of an intraperitoneal administration of matrine in CIA rats. We found that matrine could significantly mitigate joint swelling, the degree of bone erosion, and destruction in CIA rats as assessed by measuring the amount of ankle swelling, AI score, X-ray, and HE staining. We found that the therapeutic effect of matrine on CIA rats was equivalent to that of MTX, similar to the findings reported by Yang et al. [28]. In addition, an increase in the dose of matrine was associated with a corresponding enhanced therapeutic effect on CIA rats. This finding is consistent with the research results reported by Pu et al. [10], suggesting that matrine has the potential to become a novel treatment for RA.

It has been established that bone marrow suppression is a major adverse reaction associated with MTX; however, it remains unknown whether matrine can suppress the bone marrow of CIA rats. Our study is the first preliminary report of the suppressive effect of matrine on the bone marrow. No obvious inhibitory effect was observed in the bone marrow of the CIA rats in the 25 mg/kg, 50 mg/kg, or 100 mg/kg matrine groups suggesting that matrine has a high safety profile in terms of bone marrow suppression. However, whether matrine is associated with hepatotoxicity, alopecia, gastrointestinal discomfort, or other adverse reactions requires further study.

Decreased bone mass and bone destruction around the joints represent the main causes of disability in RA patients. Osteoclasts play an indispensable role in regulating bone metabolism [2]. Chen et al. [30] found that ribosomal protein S5 (RPS5) and M19, a derivative of matrine, could synergistically inhibit osteoclast formation. Furthermore, other studies have shown that M54, another derivative of matrine, could also target RPS5 and inhibit osteoclastogenesis [31]. Thus, the study of the mechanism of osteoclast activation has become an important breakthrough for the identification of potential therapeutic targets for RA.

The RANKL/RANK/OPG system is an extremely important pathway for the regulation of osteoclast function [32]. RANKL can bind to RANK and subsequently to TRAF6, located downstream, activate NF-*κ*B, and promote c-fos gene expression, which can further bind and interact with NFATc1 to induce osteoclast differentiation [33]. Our previous studies have confirmed that matrine may affect RANKL-induced osteoclast differentiation in vitro by inhibiting TRAF6, c-fos, and NFATc1 expression in a dose-dependent manner [12]. OPG mainly inhibits the activity of the RANKL/RANK signaling pathway by competing with RANK on the surface of osteoclast precursor cells to bind RANKL [3, 34]. Furthermore, changes in the OPG/RANKL ratio have been shown to directly affect osteoclast differentiation and activation [35]. Therefore, this study was the first to explore the regulatory effect of matrine on the level of OPG, OPG/RANKL, and RANKL expression in CIA rats. Compared with the healthy group, RANKL expression was significantly increased, whereas OPG and OPG/RANKL were significantly decreased in the CIA control group. This further confirmed that there was a disordered RANKL/RANK/OPG system during RA development. Following treatment with different doses of matrine, the degree of joint swelling and bone destruction was significantly decreased in the CIA rats. Moreover, the level of OPG and OPG/RANKL increased and RANKL expression decreased in a dose-dependent manner. This suggests that matrine may reduce the level of articular inflammation in CIA rats by affecting the level of RANKL and OPG expression. These results are roughly consistent with our hypothesis.

Our results demonstrate that matrine can relieve the degree of bone destruction and increase OPG expression and the ratio of OPG/RANKL, as well as decrease RANKL expression in CIA rats. Thus, matrine may represent a new drug for RA treatment; however, whether matrine can inhibit osteoclastogenesis by affecting the OPG and RANKL expression remains to be further studied.

## Figures and Tables

**Table 1:** Changes in weight gain (%) in each of the different groups before and after treatment.

Weight gain (%)								
Weight gain (%)	Healthy	Control	25 mg/kg	50 mg/kg	100 mg/kg	MTX	*F*	*P* value
Before treatment (3 w)	42.62 ± 4.83	27.79 ± 7.98^a^	29.30 ± 6.82^a^	29.22 ± 7.20^a^	25.71 ± 5.53^a^	27.61 ± 2.18^b^	4.799	0.004
After treatment (9 w)	81.93 ± 10.44	60.79 ± 12.11^a^	51.63 ± 9.25^a^	64.50 ± 11.03	66.48 ± 7.84	63.63 ± 5.15	5.143	0.003

Healthy: healthy group; control: CIA control group; 25 mg/kg: low dosage matrine group; 50 mg/kg: medium dosage matrine group; 100 mg/kg: high dosage matrine group; MTX: 2 mg/kg methotrexate group. Compared to the healthy group, ^a^*P* < 0.05; ^b^*P* < 0.01.

**Table 2:** Effects of matrine on changes in the AI score for arthritis in the CIA rats.

AI score								
AI score	Healthy	Control	25 mg/kg	50 mg/kg	100 mg/kg	MTX	*F*	*P* value
Before treatment (3 w)	0 ± 0	7.60 ± 3.647^a^	7.2 ± 1.923	7.40 ± 1.949	7.67 ± 1.528	7.80 ± 0.447	11.584	≤0.001
After treatment (9 w)	0 ± 0	11.00 ± 3.082^a^	3.8 ± 2.864^b^	1.00 ± 1.732^b^	0.67 ± 1.155^b^	1.4 ± 2.609^b^	16.276	≤0.001

Healthy: healthy group; control: CIA control group; 25 mg/kg: low dosage matrine group; 50 mg/kg: medium dosage matrine group; 100 mg/kg: high dosage matrine group; MTX: 2 mg/kg methotrexate group. Compared to the healthy group, ^a^*P* < 0.001; compared to the control group, ^b^*P* < 0.001.

## Data Availability

The data used to support the findings of this study are available from the corresponding authors upon request.
